# Celiac disease-on-chip: Modeling a multifactorial disease in vitro

**DOI:** 10.1177/2050640619836057

**Published:** 2019-03-14

**Authors:** Renée Moerkens, Joram Mooiweer, Sebo Withoff, Cisca Wijmenga

**Affiliations:** 1 Department of Genetics, University Medical Center Groningen, University of Groningen, Groningen, the Netherlands; 2 K.G. Jebsen Coeliac Disease Research Center, Department of Immunology, University of Oslo, Norway

**Keywords:** Celiac disease, complex diseases, organ-on-chip, hiPSCs, human induced pluripotent stem cells, microfluidic devices

## Abstract

Conventional model systems cannot fully recapitulate the multifactorial character of complex diseases like celiac disease (CeD), a common chronic intestinal disorder in which many different genetic risk factors interact with environmental factors such as dietary gluten. However, by combining recently developed human induced pluripotent stem cell (hiPSC) technology and organ-on-chip technology, in vitro intestine-on-chip systems can now be developed that integrate the genetic background of complex diseases, the different interacting cell types involved in disease pathology, and the modulating environmental factors such as gluten and the gut microbiome. The hiPSCs that are the basis of these systems can be generated from both diseased and healthy individuals, which means they can be stratified based on their load of genetic risk factors. A CeD-on-chip model system has great potential to improve our understanding of disease etiology and accelerate the development of novel treatments and preventive therapies in CeD and other complex diseases.

## Introduction

Approximately 0.6% to 1%^1^ of the Caucasian population has celiac disease (CeD), a complex immune-mediated disease characterized by a strong inflammatory reaction to dietary gluten in genetically predisposed individuals. CeD is a multifactorial disease caused by many genetic and environmental risk factors. In addition to gluten, viral infections^2,3^ and gut microbiome dysbiosis^4^ may also trigger disease onset. Although CeD is primarily characterized by damage to the small intestine, patients can also suffer from extraintestinal manifestations such as anemia, osteoporosis and ataxia.^5,6^ The large variation in presentation of symptoms leaves many patients undiagnosed.^7,8^ After diagnosis, the only treatment is lifelong adherence to a gluten-free diet, which can reduce quality of life^9^ and may not totally prevent gluten exposure because of “hidden” sources of gluten or cross-contamination of food products.

To better understand the natural course of CeD and design new preventive and treatment strategies, it is imperative to develop sophisticated systems that recapitulate and model the disease. Such systems have not been available thus far, but with recent molecular and technological advances—specifically in human induced pluripotent stem cell (hiPSC) technology, differentiation protocols and organ-on-chip devices—these complex modeling systems are now within reach. In this review we illustrate the complexity of CeD and describe how state-of-the-art stem cell and organ-on-chip technology can provide an in vitro model for CeD.

### Pathogenesis of CeD

#### Immune response to gluten

**Figure 1.**
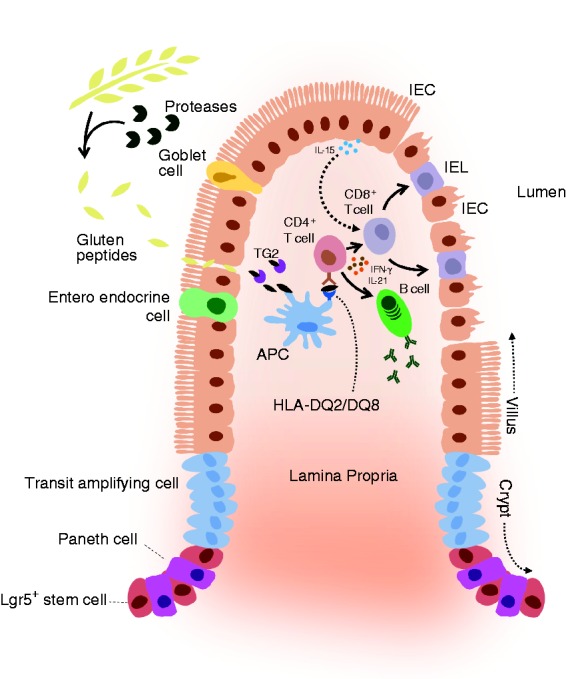
Schematic overview of celiac disease (CeD) pathobiology. Dietary gluten peptides pass the epithelial barrier, where they become deamidated by tissue transglutaminase 2 (TG2). The deamidated gluten peptides are taken up by antigen presenting cells (APCs) and are presented to CD4^+^ T cells, exclusively in the context of human leukocyte antigen (HLA)-DQ2 or HLA-DQ8. Upon gluten presentation, CD4^+^ T cells produce, among other things, interleukin (IL)-21 and interferon-gamma (IFN-γ). This leads to gluten-specific antibody production by B cells and, in concert with IL-15 production by intestinal epithelial cells (IECs), activation of intraepithelial lymphocytes (IELs), which attack the IECs, leading to villous atrophy.

The main trigger of CeD-associated inflammation is dietary gluten, a storage protein present in wheat, barley and rye. Gluten proteins are rich in glutamine and proline residues that are difficult to digest.^10^ As a consequence, incompletely digested gluten peptides pass the epithelial layer of the small intestine^11^ and enter the lamina propria where the peptide fragments are deamidated by tissue transglutaminase 2 (TG2; Figure 1). Deamidated gluten peptides have a higher affinity to class II human leukocyte antigen (HLA)-DQ2 or -DQ8 molecules on antigen-presenting cells (APCs).^12,13^ APCs presenting deamidated gluten peptides strongly activate gluten-specific CD4 + T cells, which further elicit the pro-inflammatory response characteristic of CeD. This response drives B cell-mediated generation of TG2- and gluten-specific antibodies that are used to diagnose CeD,^13^ and licenses CD8 + intraepithelial lymphocytes (IELs) to kill intestinal epithelial cells (IECs) leading to villous atrophy.^14^ Key cytokines in these processes are interferon-gamma,^12^ interleukin (IL)-15^14^ and IL-21.^15^

#### Genetic factors

Today, approximately 50% of the heritability of CeD can be explained by 45 genetic risk factors (Ricaño-Ponce et al., manuscript in preparation). The major genetic risk factors for CeD development are specific variants of the HLA class II genes (HLA-DQ2.5, HLA-DQ2.2 and HLA-DQ8), and carriership is essential but not sufficient to trigger the disease.^16^ Genome-wide association studies (GWAS) have identified 44 non-HLA risk factors, many of which are shared with other immune-related diseases (e.g. type 1 diabetes, rheumatoid arthritis, ulcerative colitis and Crohn disease).^17,18^ Most of these risk factors point to genes involved in immune response and are expressed in different types of immune cells.^19^ However, a subset of the genes are expressed in the intestinal barrier,^20^ suggesting that barrier dysfunction plays a role in CeD.

#### Environmental factors and the microbiome

Because not all carriers of genetic risk for CeD manifest the disease, non-genetic environmental factors apart from gluten may also play a role in disease onset. One such environmental factor might be the amylase trypsin inhibitors (ATIs) present in gluten-containing grains, because these can trigger a Toll-like receptor 4-dependent innate immune response in the small intestine.^21^ Additionally, viral infections (by rotaviruses, adenovirus, enteroviruses and hepatitis C virus) are associated with increased incidence of CeD.^22,23^ Interestingly, a significant number of CeD-associated genetic loci harbor transcription factor binding elements for gene products of the Epstein-Barr virus, indicating one way that viruses can regulate CeD-associated pathways.^3^ One of the few published experimental studies showed that reovirus infections can disrupt tolerance to gluten and other food antigens in HLA-DQ8–expressing mice.^2^

Furthermore, the gut microbiome composition is altered in CeD patients,^24–26^ which could be due to genetic and environmental factors. On the one hand, the HLA-DQ2 genotype introduces a selective pressure on the developing intestinal microbiome in infants.^27^ On the other, a gluten-free diet changes the microbiome composition of the intestine both in healthy adults and adult CeD patients.^28,29^ These changes in gut microbial composition can directly affect processing of gluten peptides.^30,31^ For example, CeD-associated bacteria can produce shorter gluten peptides that more easily translocate across the intestinal epithelial barrier, or modify peptides so that they activate gluten-specific T cells.^4^ Additionally, changes in the gut microbiome induced by other environmental factors (such as antibiotic use, intestinal infections and cesarean delivery) may indirectly contribute to CeD.^25^ Whether the microbiome is cause or consequence in CeD and how dysbiosis of the microbiome contributes to CeD are not clear.

#### Role of the intestinal barrier in CeD

It has been suggested that intestinal barrier function is altered in CeD,^32–34^ but it has been a matter of debate whether destruction of the barrier is only a consequence of the inflammatory immune response, or whether there is a primary defect in barrier function that contributes to disease development.^11^ Several observations, including genetic associations,^18,20^ suggest a primary barrier defect. CeD patients as well as their relatives have a higher lactulose:mannitol ratio in their urine after intake of this sugar solution when compared with control individuals and patients with aspecific gastrointestinal symptoms.^32^ It has also been reported that the morphology of tight junctions is altered in the epithelial barrier of children with active CeD, and this is only partly restored on a gluten-free diet.^35^ This is consistent with a report describing altered expression and localization of epithelial tight junction proteins in CeD patients on a gluten-free diet.^36^ Lastly, quantitative measures of barrier function, such as transepithelial electrical resistance (TEER), are decreased in biopsies of active CeD patients compared with healthy individuals, and this was only partially restored on gluten-free diet.^11^

### Current models for CeD

To date, there is no model system that fully recapitulates the complexity of CeD. Current in vitro models include immortalized cell lines and mucosal biopsies. The immune system has been investigated using cell lines of monocytes, such as THP-1, or intestinally derived T cells.^37,38^ Existing data on epithelial barrier function are largely based on intestinal mucosal biopsies or Caco-2, a tetraploid human colonic epithelial cancer cell line. Immortalized cell lines do not represent the genetics of CeD and have poor genomic integrity (Table 1). Patient-derived intestinal biopsy material does contain the CeD-associated genetic background and directly reflects the disease phenotype, but is scarce because of its invasive nature. Biopsies also have limited proliferative capacity, and individual cell types are difficult to study within a heterogeneous biopsy. Conventional systems to measure barrier function and transport, like transwell systems, do not recapitulate the intestinal physiology (e.g. IECs fail to form villus-like structures or produce mucus), and co-cultures with microbial cells are difficult in these static systems because of rapid overgrowth and contamination.^39^ Studying CeD in vivo is dependent on humanized mouse models that express human HLA-DQ8 or HLA-DQ2.^40–42^ These models have shown that the presence of gluten-specific CD4 + T cells is not sufficient to induce CeD-like pathogenesis and that triggers of the innate immune system, particularly IL-15 overexpression, are essential for inducing intestinal damage upon gluten exposure. However, mice are not ideal models because of differences in intestinal tract physiology,^43^ immune system^44^ and microbiome composition.^45^ To further elucidate the mechanisms underlying CeD, it is essential to capture the entire pathobiology of CeD using multicellular and human-based models.

**Table 1.** Possible biological systems for modeling complex diseases: advantages and disadvantages.

Biological system	Critical factors for modeling complex diseases					Other advantages (+) and disadvantages (–)
	Patient genetics	Availability of material	Genetic engineering	Heterogeneity/ complexity	Can be combined with other cell types from same donor^a^	
Immortalized cell lines	No	High	Established	Single cell type	No	+ Potentially easier to handle − Poor genomic integrity
Intestinal biopsy	Yes	Low^b^	Difficult	Multiple cell types	Yes	+ Disease phenotype − Inflamed tissue − Limited lifespan
Humanized mouse models	No	High	Established	Multiple cell types	Yes	+ Whole organism: presence of hormone, neurologic, and metabolic signals from other organs or cell systems − Requires thorough understanding of induction of disease − Difficult to translate to humans because of interspecies differences in physiology, pharmacology and cellular processes
Induced pluripotent stem cells	Yes	High	Established	Single cell type	Yes	+ Suitable for genotype selection (patient and control cases) − Requires knowledge of differentiation to relevant tissue

a Providing identical genetic background.

b Invasive procedure necessary, extremely limited “healthy” control samples.

## Novel technologies that allow CeD-on-chip

Novel advances in human stem cell biology and microfluidics technology now allow for the development of in vitro model systems with the desired genetic background, environmental factors, and interaction between disease-relevant cell types under physiological conditions.

### hiPSC and organoid technology

hiPSCs can be generated from different types of somatic cells taken from any donor. hiPSCs can divide indefinitely and have the potential to differentiate into any of the cell types found in the human body. In 2006, Yamanaka and colleagues demonstrated for the first time that human and mouse fibroblasts could be reprogrammed to a pluripotent state, resembling embryonic cells in culture.^46,47^ Pluripotency was achieved by viral overexpression of only four transcription factors: Oct4, Sox2, Klf4 and c-Myc. With the development of improved protocols, hiPSC lines can now be efficiently generated from urine-derived epithelial cells and blood-derived erythroblasts, among others.^48^

Using knowledge on embryonic development, hiPSCs can be differentiated into human intestinal organoids (HIOs): miniature parts of the gut that are cultured in a dish. The first HIOs were grown from intestinal crypts derived from human biopsy material.^49^ When cultured in an extracellular matrix (ECM) gel in the presence of specific growth factors, it is possible to maintain the stem cell niche and the proliferative and differentiation capacity of crypt cells in vitro, allowing them to grow out into complex three-dimensional (3D) “budding” structures. These structures contain multiple functional IEC subtypes that can be kept in culture for prolonged periods of time.^50^ The generation of HIOs from hiPSCs is more complex and leads to a less mature differentiated phenotype.^51^ However, embryonal development of intestinal tissue can be mimicked by exposing hiPSCs to a series of specific growth factors in a strict time-dependent manner.^52^

**Figure 2.**
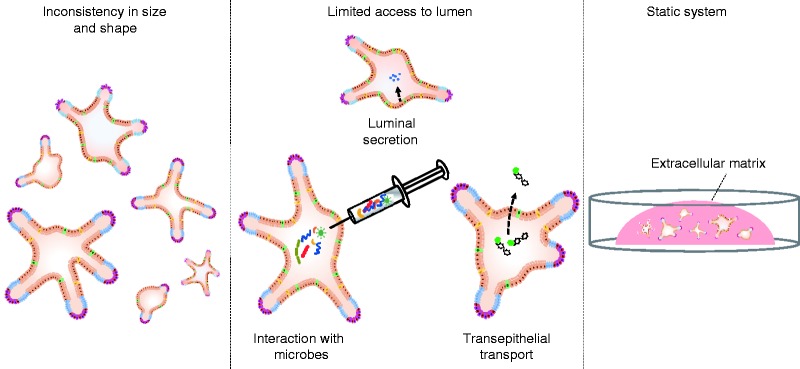
Limitations of the intestinal organoid system. Intestinal organoids are inconsistent in size and shape, which introduces variability in the results (see left panel). The closed configuration makes it technically challenging to access the lumen (apical side) of the organoids. This limits studies into interactions between intestinal epithelial cells and micro-organisms (such as commensal microbes or pathogens), studies into transepithelial transport (e.g. fluorescein isothiocyanate-dextran translocation as a measure of intestinal permeability) and analysis of luminally secreted components (see middle panel). Intestinal organoids are cultured in a static three-dimensional system as they are embedded in an extracellular matrix, which does not reflect the dynamic environment of the human intestine (see right panel).

The HIO system still has limitations when it comes to studying multifactorial diseases.^53,54^ HIOs are inconsistent in size and shape and are cultured in a static system (embedded in extracellular matrix) that does not recapitulate the intestine's physical environment (including fluid flow and peristaltic movement). The closed configuration of HIOs renders them less ideal for studying transport over the intestinal barrier or interactions with commensal microbes or pathogens (Figure 2). Apical access can be achieved by microinjection,^55^ but this technique is labor intensive and technically challenging. The wide range of organoid sizes complicates this procedure even more and makes it nearly impossible to standardize the cell:stimulus ratio. Additionally, dead cells accumulate in the enclosed lumen of the HIO, ultimately impairing the viability of the system. Lastly, physiological interactions with other components of the intestine (e.g. immune and vascular system) are difficult to emulate within the extracellular matrix, while the matrix is necessary to generate and maintain HIOs. These limitations can be overcome by an organ-on-chip system.

### Intestine-on-chip

**Figure 3.**
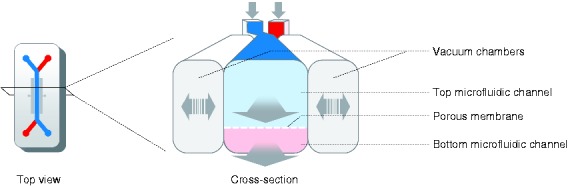
Schematic presentation of a microengineered intestine-on-chip. Intestine-on-chip systems often consist of a top microfluidic channel, resembling the gut lumen, and a bottom microfluidic channel, resembling the lamina propria and vasculature. The channels are separated by a porous membrane on which epithelial cells can be seeded and are flanked by vacuum chambers to simulate peristalsis-like movements. Unidirectional fluid flow through the microfluidic channels and contractions of the vacuum chambers simulate the physical microenvironment of the human intestine. The intestine-on-chip presented here is based on the design of Emulate Inc, Boston, MA, USA.

Organ-on-chip systems are microfluidic devices in which cells are cultured in continuously perfused microchannels engineered to mimic the physical microenvironment of tissues and organs.^53^ A current model makes use of a chip containing two parallel hollow channels approximately 1 mm wide separated by a porous ECM-coated membrane^56^ (Figure 3). In this device, a monolayer of IECs can be grown on the upper surface of the membrane separating both channels, while endothelial cells can be grown on the other side, representing blood vessels. The culture media for the cells is delivered via the upper and lower channel, which can also be used to introduce metabolites, cytokines, microbial cells and/or immune cells into the system. The system also provides mechanical forces to simulate the physical microenvironment of the intestine through fluid flow that introduces shear stress on the cells and two vacuum compartments on the sides that create a peristalsis-like motion. Remarkably, these mechanical forces induce epithelial cells to spontaneously form polarized 3D villus-like structures that contain cells expressing markers characteristic of differentiated IECs (i.e. adsorptive enterocytes, mucus-producing goblet, Paneth and enteroendocrine cells).^39,57–59^ The resulting epithelial layer exhibits basic functional properties, such as mucus production, high barrier resistance, activity of brush border and drug-metabolizing enzymes, and high efficiency in nutrient uptake because of the increased intestinal surface. These characteristics allow for studies focusing on digestion and nutrient uptake, barrier integrity and drug metabolism,^39,57,58^ and for co-cultures with commensal microbial cells for extended periods of time (up to weeks).^39,60^

In accordance with the morphological changes, the transcriptional profile of epithelial cells cultured in the dynamic chip system is very different from that of cells cultured in static Transwell systems or compared with HIO. In fact, the intestine-on-chip profile most resembles the profile of the corresponding in vivo intestinal segment.^58,60^

The material most often used for chip fabrication, polydimethylsiloxane, is fully transparent, making the chip readily amenable to microscopy. For research purposes, sophisticated intestine-on-chip systems can be engineered to contain sensors, for example to measure TEER.^61^ Integrated sensors are a major step forward because they allow for continuous monitoring of the system, something that is very difficult and laborious in conventional culture systems.

### hiPSCs, HIOs and intestine-on-chip to model CeD

**Figure 4.**
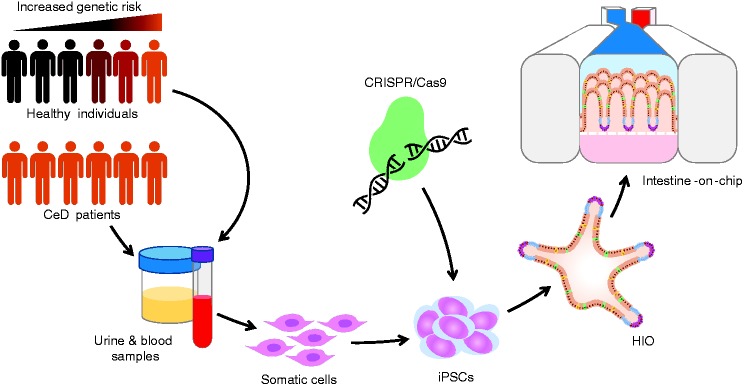
The steps from patient or healthy individual to human induced pluripotent stem cell (hiPSC)-derived intestinal organoids and intestinal barrier-on-chip. The large population and patient biobanks that have been constructed worldwide contain genomic data and stored biological material, which allow for the selection of patient and healthy control material based on genetic makeup. hiPSCs can be derived from stored kidney epithelial cells from urine or from erythroblasts from stored peripheral blood mononuclear cell fractions. These materials are obtained in a minimally invasive manner. The hiPSC lines can then be differentiated into human intestinal organoids (HIOs), which can subsequently be seeded on a microfluidic intestine-on-chip system to form an intestinal-barrier-on-chip. Specific genetic factors can be studied by genetic engineering of hiPSCs using CRISPR/Cas9 technology. For example, CeD-associated risk alleles can be reverted to protective alleles.

In contrast to monogenic diseases in which a single gene is involved, genetic modeling of complex diseases like CeD requires the inclusion of the many disease-associated genetic risk factors that need to be studied in the disease-relevant cell or tissue.^19^ Combining hiPSC and HIO technology, in vitro models of the intestine can be created from cells that contain the spectrum of CeD-associated genetic risk factors (Table 1). Because hiPSC lines can be generated from relatively easily accessible somatic cells such as urine-derived epithelial cells, skin-derived fibroblasts or blood-derived erythroblasts,^48^ there is no dependency on intestinal biopsy material obtained by invasive endoscopic procedures (in the case of CeD). This facilitates the collection of starting material from both patients and healthy individuals. Varied genetic backgrounds can then be studied to contrast the disease genetic background with low risk backgrounds (Figure 4). To study specific elements of the disease process, like barrier function, genetic engineering can be used to perturb the system by creating extreme genotypes (i.e. gene knock-out by CRISPR/Cas9 technology). These technologies could be used to generate isogenic hiPSC lines that contain identical genetic background, except for, for instance, one repaired CeD-associated genetic risk factor. Such isogenic lines may reveal the functional consequences of a single genetic variation associated with CeD. Using hiPSCs as a starting point, the effect of a disease-associated genotype can be evaluated in multiple disease-relevant cell types, either individually or in combination, in an intestine-on-chip. This model is unique because it integrates (1) the CeD-associated genetic background, (2) the interaction between disease-relevant cell types, (3) any relevant environmental stimuli and (4) the physical microenvironment of the intestine in a complex yet controllable manner. Very recently, proof-of-concept was provided for an hiPSC-derived intestinal epithelial-layer-on-chip.^59^ This system now needs to be adapted toward a more CeD-relevant model that includes hiPSC-derived endothelial^62^ and immune cells.^63–65^

## Future outlook

### Improved understanding of CeD etiology

**Figure 5.**
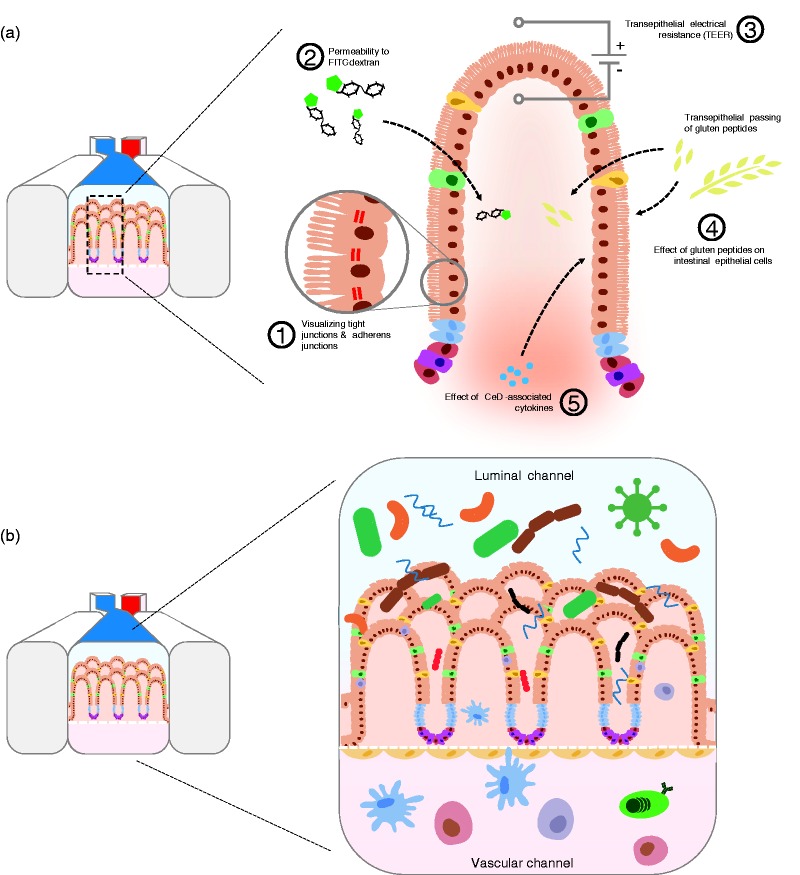
Research opportunities using a human induced pluripotent stem cell (hiPSC)-derived intestine-on-chip. (a) Functioning of the intestinal epithelial barrier in patients with celiac disease (CeD) can be assessed with the intestine-on-chip system by performing different assays: (1) Tight junctions and adherence junctions can be labeled and visualized on-chip using microscopy. (2) Barrier permeability can be assessed by measuring transepithelial passing of fluorescein isothiocyanate (FITC)-dextran complexes. (3) Barrier integrity can be tested by incorporating electrodes on-chip to measure transepithelial electrical resistance (TEER). (4) The passing of gluten peptides across the barrier and the direct effect of gluten peptides on the intestinal epithelial cells can be analyzed. (5) The effect of CeD-associated cytokines on the barrier can be analyzed by introducing the cytokines at the basolateral side (bottom channel). (b) Integration of gut microbiome, endothelial cells and immune cells in the intestine-on-chip. hiPSC-derived epithelial layers-on-chip can be extended with microbiomes from CeD patients or healthy controls on the apical side to assess the interactions between the epithelial layer and bacteria. hiPSC-derived endothelial cells can be introduced at the basolateral side to mimic the vascular system. Additionally, peripheral blood mononuclear cell (PBMC)- or hiPSC-derived immune cells can be introduced at the basolateral side to mimic the immune system.

A CeD intestine-on-chip model can help address significant questions. It will allow the investigation of the interaction between IELs and IECs in the presence or absence of triggering environmental factors. In particular, the IL-15 expression by IECs implicated in activation of IELs^14^ can be monitored in response to these different stimuli. A possible primary defect in intestinal barrier function, which in turn alters gluten transport, can be addressed using different assays in a simple system in which iPSC-derived IECs are present outside the immune context (Figure 5(a)). With this system, genes involved in the process can be identified. The role of the gut microbiome in CeD pathogenesis can also be studied. One can envision that the microbiota affects barrier function, but also that CeD-associated genetics affect microbiome homeostasis by altering the immune response. Finally, the effect of different environmental factors can be studied by introducing them into the system, for instance introducing viral ligands, metabolites produced by CeD-associated microbiota, or ATIs. The complexity of the system can be also adjusted to fit the research question, ranging from one cell type to more complex systems (Figure 5(b)).

### Development and testing of novel treatments

A lifelong adherence to a gluten-free diet has a profound impact on everyday life, which makes treatments to inhibit the strong pro-inflammatory immune response to gluten very valuable. A physiologically relevant CeD intestine-on-chip model can be used to test novel drug candidates and existing drugs for repositioning. By using patient-derived hiPSCs, differences in genetic background that may affect drug efficacy can be taken into account. To be used for drug screening and/or addressing pharmacogenetic questions, high-throughput systems should be developed, as current devices are still low throughput and costly.^66^ Nevertheless, an intestine-on-chip has great potential for personalized medicine, providing a model that can include an individual's genetic background, relevant cell types and environmental triggers.

### Toward a patient-on-chip

Although CeD is regarded as a disease of the intestine, the disease presents systemically.^6^ To capture the extraintestinal phenotypes, different organ-on-chip systems could be coupled in the future. In the context of CeD, a first expansion might be to couple an intestine-on-chip to a brain-on-chip. The intestine-brain-axis is of particular interest because the clinical spectrum of CeD includes behavioral changes such as anxiety, depression and fatigue.^67,68^ The mechanism underlying this “cross-talk” between intestine and brain is poorly understood, but proposed explanations include the interaction of gluten peptides with endorphin receptors in the brain, the migration of activated immune cells to the brain^69^ and detrimental effects of circulating microbial metabolites^70,71^—all processes that could be tested by linking organ-on-chip systems.

## Conclusion

The development of a CeD-specific intestine-on-chip model that closely recapitulates human intestinal physiology will enable in vitro studies of CeD etiology in a near in vivo situation. This will yield new insights into the role of genetic and environmental factors in CeD and may accelerate the search for novel treatments. Because genetic differences among CeD patients could be taken into account in the development of novel treatments, the efficacy of a treatment could be more accurately predicted for each individual. Moreover, this technology may improve diagnostic capacity by identifying new diagnostic markers for individuals at high risk for CeD.
